# Supplementary material for: Emotional responses to COVID-19 stressors increase information avoidance about an important unrelated health threat
Source: PLoS One. 2023 Sep 14;18(9):e0286712. doi: 10.1371/journal.pone.0286712 (PMC10501669; doi:10.1371/journal.pone.0286712)
Supplement: Attachment — Submitted filename: reviewPLOS1.docx [file pone.0286712.s001.docx]

reviewPLOS1_covidWideHealthDecidions

The manuscript presents two surveys (custom and a national survey) in which respondents who experienced higher stress in the form of income loss and local case counts felt more hopelessness, and hopelessness was associated with avoiding health information and delaying healthcare.

The manuscript is generally well-written, though some sections repeat what was previously presented. The N is large and being a representative sample makes the finding stronger. I liked the manuscript; I think it adds to the literature. The only major concern is the language of causality in a non-experimental design, but this can be easily remedied.

Some comments:

The title needs to be more specific, addressing avoidance of health information and healthcare utilization.

Under abstract:

Substitute the word "lead" to a non-causal word.

Mental health is more than hopelessness, so indeed be specific and drop 'mental health'.

The fact that there are 2 separate studies is not well-conveyed in the abstract. State explicitly that there are 2 separate studies.

Under introduction.

The statement "Information shapes beliefs and affects private behavior" needs to be supported by other references than #36 and 37, which do not examine this statement.

The authors should explain why they examined accessing treatment for non-COVID health problems as one of the DVs, an already-documented issue. Were they trying to replicate findings? To show the same findings using a big sample? To examine the process/mediators? Please present the rationale.

Structure of writing: LL 162-189 report results, and this is the introduction. The results have no place in the introduction section.

Remove words of causation or prediction from the manuscript. As most measures were taken at same or equivalent times, the word 'predicted'/'predictor' or 'increase' or 'effect' should be replaced with words of association (l. 164, 262, and elsewhere).

Please provide a DAG (=directed acyclic graph) figure conceptualizing the relationship between all variables in the study.

Under methods.

Structure each survey under respondents, materials, procedure, design. The writing is not structured enough.

Explain why a four-category variable of hopelessness made into a 2-category variable.

Streamline the analysis section. The rationale is repeated here after being presented above. There is no need for this.

On l. 312 and 341 remove the word "impacts". No experiment was performed. This is a cross sectional survey and variables have no impact on each other.

2.2.1 – it seems to me the analyses are only on the ARM study, so it's unclear why the title refers also to HPS21. HPS21 is explained under 2.2.2. The national survey appears first under 2.2.1 on l. 333 as an outcome, but this is unclear how it can be an outcome in the costumed survey.

Under Results.

The descriptive data is very interesting, especially the variance between surveys.

Pls substitute 'effect' with association in l. 472. Also impact in l. 488 and 496. The same re 'impact' all over the results.

The authors engage in a lot of interpretation in the results section, and may consider reserving it for the discussion (for example, ll. 503-508).

Table 8 is not very clear in terms of what happens with each stressor; it seems both independent variables were lumped together. A graphic representation could be helpful.

In Table 10, the difference between model I and II, and model III and IV is not clear; one surmises it's the inclusion of demographic variables, but it should be explicitly stated.

Under discussion.

Compare the variables of foregoing medical care to international data, preferably to high resource countries where medical care is less a matter of out-of-pocket payments than in the US. Delayed care was documented in many countries, but the levels may vary. There is such data, and it may increase the claim that the emotional response may drive the decision and behavior and not income issues.
