# Supplementary material for: Emotional responses to COVID-19 stressors increase information avoidance about an important unrelated health threat
Source: PLoS One. 2023 Sep 14;18(9):e0286712. doi: 10.1371/journal.pone.0286712 (PMC10501669; doi:10.1371/journal.pone.0286712)
Supplement: Attachment — Submitted filename: Response to Reviewer.docx [file pone.0286712.s003.docx]

Reviewer #3: I think that the framing of the manuscript as a study of decision-making is too broad. My recommendation would be to narrow the scope to information avoidance and remove the mention of decision making and situating the study in the larger field of decision making (impact of affect on decision-making or financial scarcity and decision making etc…). I don't think we can say with any confidence that the effects described in these other decision-making studies apply to information avoidance. The same goes for describing the outcome as health behavior (abstract) which will likely confuse the reader.

Not seeking healthcare is a broader and more multiply determined phenomenon than a single information avoidance decision about viewing a video about AMR. I think that lumping them together does a disservice to both. My preference would be to see the two studies split apart into two separate papers and the intro and discussions reworked to focus on the different outcome variables. That said, several of my comments assume that this will not be the case.

Response: We have removed all the material about healthcare decisions and retain only the text about the survey on antimicrobial resistance (AMR) and the decision to view or not to view information about AMR.

The most relevant literature reviews seem underdeveloped. I expected to read a more extensive summary of (1) studies that demonstrated an increase in hopelessness and other depressive symptoms during COVID, (2) determinants of information avoidance and, (3) the impact of COVID on health care seeking and justifying why we can consider this information avoidance or even avoidant behavior. The later is a real sticking point for me. We don’t have information on why people didn’t have care. Perhaps there was no care to receive. That was an issue for many during the pandemic. I appreciate that hopelessness predicted not receiving care so it seems like the pathway is similar to the AMR study, but I didn’t find enough information about the mediation model to feel reassured that there was true causal mediation taking place.

Response: The literature in point 3) is no longer relevant as the paper has been refocused to consider only AMR information avoidance. We have increased the text devoted to emotional responses to COVID, with a particular focus on hopelessness (lines 52-73). We have also added to the literature on information avoidance (lines 85-108). Additions to the text are highlighted in yellow.

I realize that a previous reviewer requested a shorter methods section and all the measures descriptions have been removed. This seems unusual but I defer to the editor. If they are added back in they could be rewritten to be clearer. I would give each construct its own mini paragraph and describe question wording and response options.

Response: We have re-incorporated text that had been moved out of the paper, and retained only the information about the AMR study. This section is markedly different and changes have been highlighted in yellow.

You might consider switching the order of paragraphs 1 and 2 on page 7 so you introduce the study design first and follow that with what people did in the survey. One thing that might help with clarity would be to describe what the participants did in the studies rather than the survey doing things to people.

Response: We have changed the order of the paragraphs based on the suggestion of the reviewer. Further, we have additionally attempted to change the language so that it describes what the participants did.

I don't think the analysis section indicates weighting/approach to using complex survey data unless that's in supplemental materials.

Response: We have removed the HPS data from the paper.

Lines 166-167 in the methods describe 4 nested models. I believe various covariates were added to the model. Please clarify this section so that the reader understand which variables were included in which models and why. These methods don't need to be reiterated in the results but I think a summary of the results and any important differences between the models could be articulated more clearly.

Response: We have added text to clarify the variations in the models. The text (lines 242-252) now reads, “Next, we examined the relationship of hopelessness to AMR information avoidance using binary logistic regression models. The outcome variable documenting AMR information avoidance was the choice to access or avoid a video about AMR in food and agriculture, which is a binary variable (=1 if the respondent chose to avoid the video about food, agriculture, and AMR; and =0 if they chose to access the AMR video). A second version added local cases of COVID-19 and income loss in addition to hopelessness to identify whether these COVID-19-related stressors directly influence AMR information avoidance behavior or only act indirectly through their effect on hopelessness. Next, we conducted a version of the regression that incorporated demographic variables. Finally, we examined AMR information avoidance behavior with hopelessness, COVID-19 stressors, and demographic characteristics as independent variables.”

It’s unusual for the results to be written in present rather than past tense.

Response: We have changed the results to be written in the past tense.

While some descriptive stats for the two samples makes sense, I didn’t understand the logic of presenting these as a comparison, especially since they aren’t compared in any inferential way.

Response: While the HPS analysis has been removed, we have included descriptive statistics from one wave of the HPS survey that overlapped with the data collection period for the AMR survey as the best available comparison for income loss and hopelessness from a larger US survey at a similar point in time. These data are reported in tables 2 and 4.

The stats are presented in various ways in the narrative OR and 95% CI, just the OR or just an approximation of the odd (e.g., 1.5 times). Could this be standardized so that the OR, 95% CI and p-value are always presented?

Response: We have adopted the reviewer’s requested format for reporting.

Could asterisks indicating p-values be added to the tables?

Response: We have added asterisks indicating p-values to the tables.

In the interest of preciseness the results only need to be reported once. For example in the following the first sentence can be dropped although the stats for COVID cases seem to be missing so that should be added. I’d do a careful edit for redundancy. " In the analysis of HPS21 data, experienced loss of income, expected loss of income, and state-level COVID-19 cases increase the odds of experiencing hopelessness. Experienced income loss (aOR: 2.42, 95% CI: 2.35, 3.50) and expected income loss (aOR: 2.75, 95% CI: 2.49, 3.04) significantly increase the likelihood that individuals experience higher levels of hopelessness.”

Response: We have gone through the manuscript to try to eliminate any redundancy.

Lines 255-260 seem to editorialize on the results and would seem better apportioned into parts that belong in the methods and the limitations.

Response: This text has been eliminated from the manuscript, as it was about results from the analysis of HPS data.
